# P07-12 Stakeholders perspectives on exercise referral schemes in Germany

**DOI:** 10.1093/eurpub/ckac095.112

**Published:** 2022-08-29

**Authors:** Inga Naber, Eriselda Mino, Sarah Klamroth, Anja Weissenfels, Wolfgang Geidl, Peter Gelius, Karim Abu-Omar, Klaus Pfeifer

**Affiliations:** Department of Sport Science and Sport - Physical Activity and Health, Friedrich-Alexander University, Erlangen, Germany

**Keywords:** exercise referral, physical activity promotion, primary healthcare, participatory research approach, physical activity on prescription

## Abstract

**Background:**

There is a growing popularity of exercise referral schemes (ERS) and they are widely implemented in nations such as New Zealand and Sweden. To this point, the German health care system (GHCS) is not utilising a structurally implemented ERS, but a research project is currently conducted to develop and test a German ERS. In the first project phase, the aim was to introduce the topic of ERS to relevant stakeholders of the GHCS and to gather their expert opinions on such a potential ERS. Further, the aim was to familiarise the stakeholders to the project and its collaborative approach in developing and testing an ERS.

**Methods:**

Semi-structured interviews were conducted with 12 relevant stakeholder of the GHCS. In each case, two researchers conducted the interviews between June to September 2019. Main topics addressed during the interviews were potential target groups for an ERS, their own role within an ERS, PA counselling and dropouts that would be expected. During the interviews, stakeholders were encouraged to sketch their ideas for how to organise an ERS on paper. In the analysis, we digitalised these sketches into flow-chart diagrams.

**Results:**

The analysis of the interviews showed that the sketches proposed innovative additions and alternative PA promotion strategies within the GHCS. The stakeholders identified barriers within the GHCS such as the rigid costing of treatments and performance measurements. Some reoccurring important core elements for an ERS in Germany were suggested: having a supportive person, implementing PA behaviour impact, utilising existing PA programs and tailoring individual PA counselling. Some stakeholders envisioned an ERS focusing on their perspectives and desired role within the ERS while others outlined ERS that largely excluded them.

**Conclusions:**

All stakeholders clearly expressed the need for collaboration to develop and test an ERS in Germany. Previous studies have been focused on factors that influence effectiveness, as uptake and adherence. In contrast, these interviews resulted in the identification of concrete barriers and facilitators from the administrative perspective within the GHCS. Different stakeholders show varying degrees of interest in being part of an ERS. This information is highly valuable for the upcoming collaborative process.

